# Associations between depressive symptoms, sexual behaviour and relationship characteristics: a prospective cohort study of young women and men in the Eastern Cape, South Africa

**DOI:** 10.1186/1758-2652-13-44

**Published:** 2010-11-15

**Authors:** Mzikazi Nduna, Rachel K Jewkes, Kristin L Dunkle, Nwabisa P Jama Shai, Ian Colman

**Affiliations:** 1 Department of Psychology, University of Witwatersrand, South Africa; 2 Gender and Health Research Unit, Medical Research Council, South Africa; 3 Department of Behavioral Sciences and Health Education & Center for AIDS Research, Rollins School of Public Health, Emory University, USA; 4 School of Public Health, University of Alberta, Canada

## Abstract

**Background:**

Psychological factors are often neglected in HIV research, although psychological distress is common in low- to middle-income countries, such as South Africa. There is a need to deepen our understanding of the role of mental health factors in the HIV epidemic. We set out to investigate whether baseline depressive symptomatology was associated with risky sexual behaviour and relationship characteristics of men and women at baseline, as well as those found 12 months later.

**Methods:**

We used prospective cohort data from a cluster randomized controlled trial of an HIV prevention intervention in the Eastern Cape Province of South Africa. Our subjects were 1002 female and 976 male volunteers aged 15 to 26. Logistic regression was used to model the cross-sectional and prospective associations between baseline depressive symptomatology, risky sexual behaviors and relationship characteristics. The analysis adjusted for the clustering effect, study design, intervention and several confounding variables.

**Results:**

Prevalence of depressive symptoms was 21.1% among women and 13.6% among men. At baseline, women with depressed symptoms were more likely to report lifetime intimate partner violence (AOR = 2.56, 95% CI 1.89-3.46) and have dated an older partner (AOR = 1.37, 95% CI 1.03-1.83). A year later, baseline depressive symptomatology was associated with transactional sex (AOR = 2.60, 95% CI 1.37, 4.92) and intimate partner violence (AOR = 1.67, 95% CI 1.18-2.36) in the previous 12 months. Men with depressive symptoms were more likely to report ever having had transactional sex (AOR = 1.48, 95% CI 1.01-2.17), intimate partner violence perpetration (AOR = 1.50, 95% CI 0.98-2.28) and perpetration of rape (AOR = 1.81, 95% CI 1.14-2.87). They were less likely to report correct condom use at last sex (AOR = 0.50, 95% CI 0.32-0.78). A year later, baseline depressive symptomatology was associated with failure to use a condom at last sex among men (AOR = 0.60, 95% CI 0.40-0.89).

**Conclusions:**

Symptoms of depression should be considered as potential markers of increased HIV risk and this association may be causal. HIV prevention needs to encompass promotion of adolescent mental health.

## Background

The HIV epidemic is continuing to expand globally as prevention efforts show limited success in many population groups [1-3]. Research from southern Africa on risky sexual behaviours and relationships in which HIV transmission may occur emphasizes social, cultural, political and economic factors to the neglect of psychological factors [4-7]. Yet psychological distress is common in low- to middle-income countries [8], such as South Africa [9-12]. Evidence that social circumstances and knowledge do not explain sexual risk behaviours alone suggests that we need to deepen our understanding of the role of mental health factors in the HIV epidemic [13-17].

Research has found depressive symptomatology among youth to be associated with earlier sexual debut, higher numbers of lifetime sexual partners, concurrent, multiple and casual sexual partnerships, substance use at last sex, pregnancy, non-use of contraception, perceived barriers to condom use and having more risky partners [16,18-28]. However, most of this evidence comes from high-income countries, and these associations have not been established in South Africa or other countries with generalized HIV epidemics.

In South Africa, there is conflicting evidence on the association of depressive symptomatology with condom use, with one study showing greater use of condoms among respondents with depressed symptoms [10] and another reporting the opposite [25]. A community-based cross-sectional study from South Africa confirmed the associations between depression and having concurrent partnerships, previous treatment for a sexually transmitted infection, sex with someone known for less than one day, sex while using alcohol or drugs, transactional sex and forced sex [10].

This study was carried out with a general adult population sample and did not disaggregate findings by age or gender, leaving open questions as to any differences between women and men, and associations among adolescents. The objective of our study was to investigate the association between baseline depressive symptomatology, and sexual behaviour and relationship characteristics both at baseline and 12 months later among young women and men in the rural parts of the Eastern Cape Province of South Africa.

## Methods

Between 2002 and 2004 a cohort of young women and men participated in the Stepping Stones Study, a cluster randomized trial to evaluate the effectiveness of a behavioural prevention intervention for HIV [29,30]. The study was conducted in 70 sites (clusters) around the small city of Mthatha. These were grouped into seven strata: an urban stratum and six rural strata. Participants were volunteers aged 15 to 26 who resided in the clusters. Participants were interviewed at baseline, invited to attend one of the two study arms and then re-interviewed 12 and 24 months later. Methodology, recruitment, characteristics of the study site and main trial findings are described elsewhere [29,30]. Ethical approval for the Stepping Stones Study was granted by the University of Pretoria and University of Witwatersrand.

All interviews were conducted by highly trained young, sex-matched interviewers to achieve more open disclosure of sexual behaviours. A standardized structured questionnaire administered in isiXhosa, the local language, was used to collect data in one-on-one, face-to-face interviews. A year later, 1002 (78%) women and 976 (76%) men were re-interviewed. The two main reasons for loss to follow up were relocation and death [30].

### The questionnaire

#### Background characteristics

The questionnaire asked about date of birth and highest school grade passed. Age in years was treated as a continuous variable. Education was dichotomized into "educated up to Grade 10 versus further". Alcohol abuse was measured using the AUDIT Alcohol Screening Test and a cut-off point of 8 indicated a drinking problem [31]. Socio-economic status was measured using questions developed for this study that captured household goods ownership (TV, radio, car), food and meat availability, and access to a modest sum of R100 (US$13 in 2010) for medical emergency. A higher score reflected greater wealth. For women, the Cronbach's alpha was 0.55, and for men 0.60.

Childhood adversity was assessed using a 23-item revised Childhood Trauma Questionnaire, which measured emotional and physical neglect, and emotional, physical and sexual abuse [32,33]. Participants were asked whether, before the age of 18, they had experienced each act never, sometimes, often or very often. A typical item read, "I was beaten so hard at home that it left a mark or bruise." A higher score reflected more trauma experienced. The Cronbach's alphas were 0.77 in women and 0.75 in men.

Participants were asked their age at first sex, referring to penetrative vaginal sex, and whether this sexual experience was willing, persuaded, tricked, forced or rape. First sexual experience was dichotomized into: "willing, persuaded and tricked" versus "forced or raped". All were asked if they had ever used a condom. Women were asked if they had ever been pregnant or were pregnant at the time of the interview, and men were asked if they have been told by any of their sexual partners that they had made them pregnant.

#### Depressive symptomatology

Depressive symptoms were measured using the Centre for Epidemiological Studies on Depression (CES D) Scale [34]. This is a common screening scale measuring feelings and behaviours characteristic of symptoms of depression during the past week. It consists of 20 items scored from rarely experienced (0) to experienced most or all of the time (3). A typical item read, "During the past week, I was bothered by things that usually don't bother me." Scores ranged from 0 to 60, and 16+ was used as a cut-off point for probable depressive caseness [34]. This tool has been previously used in South Africa for community-based research studies [10,35]. The Cronbach's alpha for women was 0.90 and 0.91 for men.

#### Outcome variables: sexual behaviours and relationship characteristics

At baseline, information about sexual behaviours and relationships was reported for lifetime or current status, and at follow up, it was recorded for the period since the last interview or in the past 12 months. Participants reported the number of main and casual sexual partners, and a total of three or more partners were used as our "high" category. Women were asked the age of their main partner and the difference between this and their own age was calculated. We considered a woman to have had an older partner if he was three or more years older [36]. We asked about correct condom use with the main partner at last sex, defined as the condom not breaking, slipping off, not put on during the sexual act, or removed prematurely.

Knowledge of partner concurrency was assessed by a question, "How likely do you think it is that (NAME OF PARTNER) is having sex with someone else - would you say definitely is, probably is, probably is not or definitely not?" Responses were dichotomized into "definitely or probably is non-monogamous" or "probably or definitely is monogamous". We asked respondents about engaging in transactional sex using questions that have been used in similar settings [37]. Transactional relationships were defined as those where material gain was the main motivating factor for the relationship or sexual encounter with a main, casual or one-night stand partner. For women, the questions were about receiving, and for men, about both giving to a woman and receiving from a woman.

Relationship control was measured on 13-item modified version of the Sexual Relationship Power Scale [38]. A typical item from the scale (answerable in "strongly agree", "agree", "disagree", "strongly disagree") was, "When (NAME OF BOYFRIEND) wants me to sleep over, he expects me to agree." For men, the reverse read, "When I want (NAME OF GIRLFRIEND) to sleep over, I expect her to agree." A higher score meant there was equitable power sharing in the relationship. The Cronbach's alpha for women was 0.68; for men, it was 0.54.

Questions on intimate partner violence (IPV) were adapted from the World Health Organization instrument; they asked women about experiencing violence and men about perpetrating violence [39]. The IPV questions inquired about specific physically violent practices: pushed, shoved, slapped, hit with fist, kicked, beaten up, strangled, burnt, hurt/threatened with a weapon, and threw something that could hurt her, as well as their frequency. Sexual IPV questions asked about forced sex or sex performed because the woman was afraid of negative consequences. Based on prior research in this setting, a variable measuring IPV exposure was derived, which dichotomized it as zero or one episode versus two or more episodes. Men were additionally considered to have raped a non-partner if they responded affirmatively to any question on individually or group-perpetrated rape of a woman who was not a girlfriend. An example of items measuring non-partner rape is, "Was there a time when you made a woman or girl, other than your girlfriend at the time, have sex with you when she did not want to?"

### Statistical analysis

Data were analyzed using StataIC 11. All analyses took into account the study design with the dataset viewed as a cohort, and a stratified, two-stage structure with participants clustered within the 70 study sites [29]. Data were analyzed separately for women and men [40]. Analysis was restricted to only those participants who had had sex at baseline, and at follow up, to those who had had sex in the previous 12 months.

The socio-demographic composition of the sample and background sexual history was analyzed for participants with depressed symptoms compared with those without the symptoms. Exposures were summarized as percentages (or means) with 95% confidence limits, using standard methods for estimating confidence intervals from complex multistage sample surveys (Taylor linearization). Pearson's chi was used to test associations between categorical variables. To account for clustering of participants within villages, random effects (multilevel) models were fitted. Analysis of the background characteristics of those participants lost to follow up showed that they were not different from those who remained in the study in terms of their baseline depression status and sexual measures (Table 1).

**Table 1:** Baseline characteristics of lost to follow up and the remaining cohort: women and men

**Variable**	**Lost to follow up** **Mean (min-max)** **Count (%)**	**Followed up** **Mean (min-max)** **Count (%)**	**p value**	**Lost to follow up** **Mean (min-max)** **Count (%)**	**Followed up** **Mean (min-max)** **Count (%)**	**p value**
	**WOMEN**			**MEN**		
	
	n = 293	n = 1002		n = 312	n = 976	
Depression	55 (18%)	218 (21%)	0.621	48 (15%)	127 (13%)	0.730
Age	18 (15-23)	18 (15-26)	1.000	19.39 (15-23)	19.13 (15-26)	**0.020**
Socio-economic status score	-0.06 (-3.06, 3.31)	-0.01 (-3.06, 3.84)	0.573	-0.11 (-2.91, 3.52)	0.03 (-2.91, 3.52)	0.139
Adverse childhoods score	0.01 (-1.28, 3.81)	0.04 (-1.28, 6.34)	0.644	0.09 (-1.29, 5.09)	-0.03 (-1.29, 9.41)	0.066
Age at first sex	15 (9-21)	15 (6-22)	0.377	14 (6-20)	14 (5-23)	0.802
Educated up to Grade 10	241 (82%)	872 (87%)	**0.048**	253 (81%)	868 (88%)	**0.004**
Alcohol problem	15 (5%)	31 (3%)	0.186	81 (25%)	256 (26%)	0.857
First sex experience was willing	75 (25%)	267 (26%)	1.000	302 (96%)	948 (97%)	0.392
Ever used a condom	168 (57%)	635 (63%)	0.154	193 (61%)	646 (66%)	0.201
Ever been pregnant	60 (20%)	231 (23%)	0.619	52 (16%)	156 (16%)	1.000

To test the hypothesis that baseline depressive symptomatology was associated with sexual risk, we built logistic regression models adjusting for study design and controlled for possible confounding variables that were chosen on the basis of literature [20,41-45]. The confounding variables adjusted for were socio-economic status, experiences of childhood adversity, alcohol abuse and education. A separate model was build for each variable.

To test the hypothesis that baseline depressive symptomatology was associated with subsequent sexual risk, we built logistic regression models that controlled for the baseline level of each variable, the intervention arm and study design. We tested for interaction between depression and intervention arm, and preset the adjusted ORs for this where the effect was more than 10%.

## Results

### Sample background and socio-demographic characteristics

Baseline prevalence of depressive symptoms was 21.1% among women and 13.6% among men. Table 2 shows the background characteristics of participants. Women and men with depressive symptoms were more likely to have suffered trauma in childhood and to be currently abusing alcohol. Additionally, women had lower socio-economic status, but were also more likely to have completed a Grade 10 education.

**Table 2:** Background demographic characteristics for women and men

	WOMEN (n = 1294)			MEN (n = 1288)		
	Depressed mood Mean (min-max) Count (%)	No depressed mood Mean (min-max) Count (%)	p value	Depressed mood Mean (min-max) Count (%)	No depressed mood Mean (min-max) Count (%)	p value
Age	18 (15-23)	18 (15-26)	0.741	19 (15-23)	19 (15-26)	0.200
Educated up to grade 10	245 (89%)	868 (85%)	**0.046**	155 (88%)	966 (86%)	0.545
Alcohol use problem	19 (6%)	27 (2%)	**0.001**	72 (41%)	265 (23%)	**0.000**
Socio-economic status score	-0.24 (-3.06, 3.84)	.03 (-3.06, 3.84)	**0.005**	-0.08 (-2.91, 3.52)	0.01(-2.91, 3.52)	0.455
Experience of adverse childhood	0.47 (-1.28, 4.19)	-0.07 (-1.28, 6.34)	**0.000**	0.61(-1.29, 9.41)	-0.10 (-1.29, 5.41)	**0.000**
Age at first sex	15 (8-21)	15 (6-22)	0.431	14 (7-19)	14 (5-23)	0.188
Ever used a condom	179 (65%)	624 (61%)	0.249	113 (65%)	726 (65%)	0.994
Ever been pregnant	69 (25%)	222 (21%)	0.215	37 (21%)	171 (15%)	0.078

### Depressive symptoms, sexual behaviour and relationship characteristics

At baseline, women with depressed symptomatology were more likely to be in highly controlling relationships, have a partner who was three or more years older and have experienced two or more lifetime episodes of physical or sexual intimate partner violence. After adjusting for potential confounders, having depressive symptoms was associated with both experience of two or more episodes of physical or sexual IPV and having a partner three or more years older (Table 3). Women who had depressed symptomatology at baseline were more likely to have had transactional sex and have experienced two or more new episodes of IPV 12 months later, and there was a suggestion that they may have been more likely to have had a non-monogamous partner.

**Table 3:** Associations between depressive symptoms, sexual behaviours and relationship characteristics for women

	Baseline count (percentage) n = 1294						12 months later count (percentage) n = 995			
	D	ND	OR	P	AOR^1^	P	D	ND	AOR^2^	P
3+ lifetime sexual partners [AOR^2 ^past year]	104 (38%)	343 (33%)	1.21 (0.91,1.61)	0.170	0.92 (0.67,1.25)	0.603	19 (8%)	57 (7%)	1.14 (0.65, 2.10)	0.633
Transactional sex	78 (28%)	240 (23%)	1.28 (0.96, 1.71)	0.082	1.12 (0.81, 1.55)	0.487	44 (20%)	104 (13%)	**2.60** **(1.37, 4.92)**	**0.003**
More liberal power dynamics in the relationship vs. less liberal	184 (69%)	761 (77%)	.67 (0.47, 0.95)	**0.029**	0.81 (0.58, 1.13)	0.229	146 (68%)	609 (80%)	0.72 (0.42,1.23)	0.237
Partner has other partner(s)	177 (66%)	643 (64%)	1.06 (0.79, 1.40)	0.677	1.00 (0.74,1.35)	0.976	157 (74%)	506 (66%)	1.38 (0.97, 1.97)	0.073
Had a main partner who was 3+ years older	165 (61%)	526 (53%)	1.42 (1.05, 1.91)	**0.021**	1.37 (1.03, 1.83)	**0.029**	128 (60%)	442 (58%)	0.84 (0.50, 1.21)	0.371
Correct condom use with main partner at last sex	98 (35%)	365 (36%)	0.99 (0.74, 1.32)	0.960	1.08 (0.80, 1.46)	0.572	113 (54%)	386 (52%)	1.07 (0.77, 1.49)	0.658
2 or more episodes of physical or sexual abuse	127 (46%)	217 (21%)	3.22 (2.44, 4.24)	**0.000**	2.56 (1.89,3.46)	**0.000**	71 (26%)	142 (13%)	1.67 (1.18, 2.36)	**0.003**

D: depressed symptoms; ND: no depressed symptoms

OR: crude odds ratios for the association between baseline depression and baseline sexual behaviour outcomes

AOR^1^: Baseline depressive symptoms and baseline sexual behaviour and relationship characteristics adjusted for age, socio-economic status, education, alcohol, childhood adversity and study design

AOR^2^: Baseline depressive symptoms and sexual behaviour and relationship characteristics a year later adjusted for baseline level of each variable and study design

CI: 95% confidence intervals

Among men at baseline, reporting three or more lifetime partners, transactional sex, having a non-monogamous current partner, not using condoms correctly at last sex, perpetration of two or more episodes of physical or sexual IPV, and rape perpetration were associated with depressive symptoms. After adjustment for confounding, having depressive symptoms at baseline was associated with having had transactional sex, perpetrated IPV, having raped a stranger or acquaintance, and not using a condom correctly with the main partner at last sexual intercourse (Table 4). Twelve months later, men with depressed symptoms at baseline were less likely to have used a condom with a main partner at last sex. These results were similar to those found when we used the CESD scale as a continuous variable (findings not shown).

**Table 4:** Associations between depressive symptoms, sexual behaviours and relationship characteristics for men

	Baseline count (percentage) n = 1288						12 months later count (percentage) n = 984			
	D	ND	OR	P	AOR^1^	P	D	ND	AOR^2^	P
3+ lifetime sexual partners [AOR^2 ^last year]	102 (58%)	533 (47%)	1.55 (1.11,2.17)	**0.010**	1.26 (0.88, 1.78)	0.192	58 (47%)	333 (39%)	1.46 (0.82, 2.60)	0.194
Transactional sex	70 (40%)	296 (26%)	1.87 (1.34, 2.60)	**0.000**	1.48 (1.01, 2.17)	**0.040**	12 (9%)	57 (6%)	0.76 (0.26, 2.21)	0.616
More liberal power dynamics in the relationship vs. less liberal	144 (84%)	877 (82%)	1.19 (0.71, 1.99)	0.492	1.24 (0.77, 2.00)	0.362	101 (80%)	671 (82%)	0.74 (0.44, 1.23)	0.256
Partner has another partner(s)	70 (41%)	327 (30%)	1.55 (1.16, 2.08)	**0.003**	1.32 (0.93, 1.88)	0.117	40 (31%)	251 (30%)	1.09 (0.71, 1.65)	0.683
Correct condom use with main partner at last sex	29 (16%)	324 (29%)	0.48 (0.32, 0.74)	**0.001**	0.50 (0.32, 0.78)	**0.002**	35 (43%)	416 (51%)	0.58 (0.38, 0.87)	**0.010**
2 or more episodes of physical or sexual abuse	46 (26%)	150(13%)	2.28 (1.59, 3.27)	**0.000**	1.50 (0.98, 2.28)	**0.057**	24 (18%)	110 (12%)	1.29 (0.75, 2.19)	0.347
Stranger rape perpetration	35(20%)	104(9%)	2.44 (1.56, 3.81)	**0.000**	1.81 (1.14, 2.87)	**0.011**	19 (14%)	78 (9%)	1.58 (0.90, 2.77)	0.111

## Discussion

Our findings show that depressive symptoms are associated with behaviours and relationship characteristics that put young South African women and men at risk for sexually transmitted HIV. For women, having depressive symptoms was associated with having experienced one or more incidents of physical or sexual IPV and dating a partner three or more years older, as well as a greater likelihood over the following 12 months of having transactional sex and experiencing further IPV.

In men, depressive symptoms at baseline were associated with having had transactional sex, not using a condom or doing so incorrectly at last intercourse, and having raped a stranger or acquaintance; there was some evidence suggesting that they were also associated with having perpetrated more than one episode of physical or sexual IPV. Men who reported baseline depressive symptoms were at greater likelihood of not using a condom or doing so incorrectly at last intercourse 12 months later. While baseline associations can be bidirectional, and we have no knowledge of the duration of how depressive symptomatology, the associations found in the prospective analysis confirm that depressive mood predicted sexual risk 12 months later.

Intimate partner violence victimization and perpetration were associated with depressed symptoms in both the cross-sectional and prospective analyses. It is known that, for women, being in an abusive relationship causes depression [46,47], and our prospective findings show that a depressive state predicted vulnerability to abuse and to a controlling partner, a situation that is likely to generate more depressive symptoms. Addressing IPV in HIV is important as it has been shown to increase risky sexual behaviours and risk of HIV infections [33,36,47-51]. Baseline findings here measured past and current IPV, suggesting therefore that prevention of violence could have prevented baseline depressive symptoms. Evidence presented here suggests that the presence of depressive symptoms was associated with both sexual victimization of males and male rape perpetration. This is in agreement with previous research [46].

Male condoms are widely and freely available in South Africa and condom use is an act that is mainly male-controlled [15]. There are many reasons for young adults not to use condoms, including preference for sex without one, trusting a partner, a belief in the safety of a steady relationship, lack of persuasive power, relationship conflict, and, for women, gender inequity and fear of violence [14,15,52-55]. It is possible that feelings of helplessness and hopelessness associated with depression reduce men's interest in, or determination around, protective behaviour. This study shows that promoting mental health needs to be recognized as important for condom use promotion.

In this study, depressive symptomatology was associated with dating a man three or more years older for young women, and this is an important risk factor because HIV prevalence in older men in this setting is higher [36,56,57]. Important social factors increasing young women's vulnerability to HIV in relationships with older men are gender power inequalities, IPV and transactional sex [58-60]. Young women with depressive symptoms may try to please their partners, becoming unassertive and more vulnerable to abuse, especially if the primary motivation for the relationship was material gain. In the baseline analysis, it is not possible to know if depressive symptomatology preceded having older male partners or followed them. However, the study findings suggested that family-level financial hardships and dating older partners need to be addressed together in HIV prevention programming and these should integrate interventions aimed at reducing depressive symptoms.

These findings are useful in helping us understand the interactions between depressive symptoms and sexual risk. The study participants were recruited for an HIV behavioural intervention evaluation and there may have been under-reporting of sexual risk taking, but we attempted to reduce this bias by standardizing measurements, fieldworker training and most of the baseline data being collected by four interviewers. The use of different questions to measure the outcome by asking about the current or most recent partners and actions provided a robust way to maximize recall. To encourage more honest disclosure by participants, we assured them of confidentiality, communicated survey legitimacy, and ensured rapport between interviewer and respondents; these are all taken to encourage accurate disclosure in survey interviews of sexual behaviours [61]. Field workers had no knowledge of baseline depressive symptomatology when interviewing at 12 months.

## Conclusions

As HIV risk in late adolescence continues to be a serious concern in southern Africa [13,32,60], this study shows evidence that depressive symptoms predicted some sexual risk. This implies that there should be upstream interventions to prevent depressive symptoms and associated risky sexual behaviours. Though some of the associations were different for women and men, treating depressive symptoms in both sexes could result in a reduction of risky sexual behaviours and relationships.

Youth mental health services remain neglected in sub-Saharan Africa. The Lancet Global Mental Health Group issued a "call for action" in 2007 to strengthen mental health services across the globe, particularly in low-income countries, and emphasized the prevalence and burden of depressive disorders and the low cost of effective interventions [8]. Mental health investments could have an enormous impact on public health and specifically on the HIV epidemic in sub-Saharan Africa. We recommend that the existing youth-friendly sexual and reproductive health services, which are more widely available, accessible and already tackle health issues, such as teenage unplanned pregnancies and HIV prevention, should integrate prevention, diagnosis and management of depressive symptoms in youth.

## Competing interests

The authors do not have any competing interests to declare. The funding sources did not influence the outcomes of our work.

## Authors' contributions

MN, the first author of this paper, contributed to the design of the study, project management, data collection, analysis and interpretation of findings, and took a lead responsibility in writing up the paper. RJ, the project leader throughout the trial, wrote the proposal, led on the study design, intervention adaptation, and questionnaires, managed the study, directly managed the project from September 2004 to April 2006, and did much of the data management. She also supervised data analysis, data interpretation and the writing up of the paper. KD contributed to the development of the data collection tools, gave technical assistance to the project, and participated in data management and the interpretation of findings. NJS contributed to the development of data collection tools, data collection, management of the project and interpretation of results. IC supervised data analysis, data interpretation and the writing up of the paper. All authors read and approved the final manuscript.
